# Low-Intensity Exercise Attenuates Immune Checkpoint Inhibitor-Induced Cardiotoxicity via Regulation of Metabolism and Autophagy

**DOI:** 10.3390/cancers18010138

**Published:** 2025-12-31

**Authors:** Louisa Tichy, Traci L. Parry

**Affiliations:** Kinesiology Department, University of North Carolina Greensboro, 1408 Walker Ave., Greensboro, NC 27412, USA

**Keywords:** immune checkpoint inhibitors, cardiotoxicity, exercise

## Abstract

Background: Immune checkpoint inhibitors (ICIs) are a new anti-cancer therapy that have improved survival rates in many aggressive cancers. However, while rare, a significant number of patients develop ICI-induced cardiotoxicity. Clinical manifestations are non-specific and underlying cellular mechanisms remain unknown, making diagnosis and treatment of these ICI-induced cardiac side effects difficult. Exercise has shown protective effects against chemotherapy-induced cardiotoxicity but has not been investigated in combination with ICIs. High-intensity exercise has shown greatest cardioprotective effects in preclinical (animal) models, but human cancer patients prefer low-intensity exercise in the clinical setting. Therefore, the purpose of this study was to further identify the cardioprotective effects of low-intensity exercise as a treatment strategy against ICI-induced cardiotoxicity. Methods: Female mice were randomly selected and separated into four groups: sedentary (SED), sedentary ICI-treated (SED + ICI), low-intensity treadmill-exercised (TM), and low-intensity treadmill-exercised ICI-treated mice (TM + ICI). Mice either underwent a 4-week low-intensity treadmill exercise protocol (TM) or remained sedentary (SED). During the 4 weeks, ICI mice received anti-PD-1 treatment (200 μg/mouse) via intraperitoneal injections twice each week. Echocardiography was performed at baseline and sacrifice to determine changes in cardiac structure and function. At sacrifice, cardiac tissue was collected, weighed, and frozen for further biochemical analysis. Underlying metabolic signaling pathways were assessed via Western Blot, and autophagic flux was analyzed via fluorescent microscopy. Results: Echocardiography at sacrifice revealed significantly decreased fractional shortening as a measure of cardiac function (−20%), 1.5-fold dilation of the left ventricle, and thinning of the posterior cardiac wall at systole and diastole in SED + ICI mice compared to SED controls (*p* < 0.05), indicative of a phenotype of ICI-induced dilated cardiomyopathy. TM + ICI mice did not show a significant difference in these cardiac structural and functional parameters, suggesting cardioprotective effects of low-intensity exercise. In line with these findings, Western Blot and fluorescent microscopy analyses revealed upregulation of autophagic flux (*p* < 0.05), as well as dysfunctional metabolic pathways (*p* < 0.05) in ICI-treated mice compared to non-ICI controls. Low-intensity exercise was associated with regulation of dysfunctional metabolism and autophagy in TM + ICI compared to SED + ICI mice. Conclusions: The clinically relevant ICI treatment protocol used in this study led to significant cardiac dysfunction and remodeling, accompanied by underlying dysfunctional metabolism and autophagy. Low-intensity exercise was capable of regulating abnormal protein synthesis and degradation and protecting against ICI-induced cardiotoxicity. This study adds knowledge to the characterization of still unclear clinical manifestations of ICI-induced cardiotoxicity, underlying signaling pathways that could shed light on potential pharmacological treatment targets, as well as the protective effects of low-intensity exercise as a non-pharmacological treatment strategy.

## Simple Summary

Immune checkpoint inhibitors, such as anti-PD-1, are a relatively new and very effective cancer treatment, but in rare cases patients can suffer from cardiac remodeling and damage, leading to reduced cardiac function that is difficult to diagnose and treat. In this study, we found that mice treated with anti-PD-1 immune checkpoint inhibitors developed cardiac remodeling and dysfunction, while mice that performed low-intensity exercise concurrent with anti-PD-1 treatment showed preserved cardiac function and protection against abnormal cellular signaling pathways. These findings suggest that low-intensity exercise may be a safe and feasible adjuvant treatment option to help protect the heart in patients receiving immune checkpoint inhibitors.

## 1. Introduction

Immune checkpoint inhibitors (ICIs) are a relatively new (first approved by the FDA in 2011) type of anti-cancer immunotherapy that initiate a biological immune response to promote tumor cell destruction [1]. ICIs have significantly improved long-term remission of many aggressive cancers and are associated with fewer side effects compared to numerous other anti-cancer therapies (e.g., chemotherapy, tyrosine kinase inhibitors) [2]. However, despite the positive clinical outcomes, a disproportionate number of patients treated with ICIs, specifically lung cancer patients, develop ICI-induced cardiotoxicity (i.e., myocarditis, new arrhythmias, heart failure) [3] with often fatal outcomes. While initially reported incidence rates range from 0.06 to 1%, the actual incidence is likely much higher due to non-specific clinical manifestations and a lack of awareness and limited diagnosis of cardiotoxic side effects associated with ICIs [3].

While pharmacological anti-inflammatory treatments have been successful in overcoming ICI-induced cardiotoxicity in preclinical models [4], exercise is a promising non-pharmacological alternative that offers a multitude of benefits. Some of these benefits of exercise as a non-pharmacological treatment option include improved quality of life, mental health, improved anti-cancer treatment tolerance, few side effects, accessibility, and affordability, as well as anti-inflammatory and cardioprotective effects [5,6,7,8]. Exercise, defined as physical activity carried out to improve health and fitness, has been shown to attenuate cardiac remodeling and downregulate cardiac inflammation, offering protective effects against cancer-induced cardiac dysfunction and cachexia, and chemotherapy-induced cardiotoxicity [5,9,10]. Exercise of different intensities can improve immune function, regulate inflammatory pathways, and induce reductions in tumor growth [11]. Additionally, in cardiac rehabilitation settings exercise has shown significant protective effects against coronary artery disease and other cardiovascular diseases, which are risk factors in the aging cancer population [12,13]. Thus, exercise is a promising adjuvant therapy that can preserve cardiac structure and function and could aid in overcoming ICI-induced cardiotoxicity, supplying a potential path to boost long-term remission rates and decrease the severity of ICI-associated side effects. However, the most effective exercise approach (i.e., modality, intensity, duration) to optimize ICI-treatment remains unknown, hindering optimal implementation of exercise as an adjuvant therapy in cancer patients treated with ICIs.

While high-intensity exercise shows greatest cardioprotective effects in preclinical (animal) models, safety and motivational attitudes toward high-intensity exercise are of great concern in the clinical setting [14]. Based on survey data, human cancer patients choose lower intensity exercise (e.g., walking outdoors) that they can perform without medical supervision as the preferred choice of exercise [14]. However, observational studies have shown significantly decreased physical activity levels and increased sedentary behavior in cancer patients and survivors, which is associated with greater risk of chronic disease and cancer mortality [15,16,17]. Targeting low-intensity, structured exercise as a means to increase physical activity levels and decrease sedentary behavior may therefore be a promising strategy to decrease risk of treatment-related side effects and mortality and increase quality of life of cancer patients. To date, research evaluating the benefits of lower intensity exercise is limited. A small number of preclinical studies have identified protective effects of low-intensity exercise against chemotherapy-induced cardiotoxicity [18,19,20]. But, to our knowledge, research identifying the effects of low-intensity exercise on ICI-induced cardiotoxicity has not been conducted yet.

Therefore, the purpose of this study was to determine if low-intensity treadmill exercise can protect against ICI-induced cardiotoxicity in mice. Mouse model work is the crucial first step to identify manifestations of ICI-induced cardiotoxicity and the most beneficial multimodal approach prior to clinical implementation. The central hypothesis of this project was that low-intensity exercise protects against ICI-induced cardiotoxicity by preserving cardiac structure and function and regulating molecular signaling pathways in the heart of ICI-treated mice.

## 2. Methods

### 2.1. Research Design

Female LC3 transgenic (C57BL/6-Tg(CAG-RFP/EGFP/Map1lc3b)1Hill/J; Jackson Laboratory strain #027139) and age/sibling matched C57BL/6 wildtype mice (~12–14 weeks old; adulthood) were used for this study. Female mice were used due to preclinical and clinical observations that have identified increased risk of development of ICI-induced cardiotoxicity and increased mortality rates in females [21,22,23]. Mice were bred onsite in the animal facility at the University of North Carolina Greensboro. Animals were housed in standard mouse cages in a temperature-controlled facility with 12:12 light–dark cycle. All mice had *ad libitum* access to water and food, consisting of standard rodent chow diet. All procedures were approved by the University of North Carolina Greensboro’s Institutional Animal Care and Use Committee and comply with the Animal Welfare Act guidelines. Sample size was chosen based on power calculations (power, 80%; alpha: 0.05). Mice were randomly divided into 4 groups: sedentary non-ICI (SED; n = 5), sedentary ICI (SED + ICI; n = 5), low-intensity treadmill-exercised non-ICI (TM; n = 5), and low-intensity treadmill-exercised ICI (TM + ICI; n = 5). Mice either underwent a low-intensity treadmill exercise protocol (TM groups, see Low-Intensity Treadmill Protocol section) or remained sedentary (SED groups) for 4 weeks. On day 1 of week 1, animals in TM groups started the low-intensity treadmill exercise protocol, while SED mice remained sedentary. Starting in week 1, ICI mice received immune checkpoint inhibitor (ICI) treatment (see ICI Treatment section). Cardiac muscle function was assessed by conscious echocardiography at baseline and sacrifice. On day 28, mice were euthanized by isoflurane overdose followed by cervical dislocation. Cardiac tissues were collected, weighed, and either flash frozen in liquid nitrogen or embedded in optimal cutting temperature gel (OCT) and frozen on dry ice for subsequent biochemical analyses.

### 2.2. Immune Checkpoint Inhibitor Treatment

The PD-1 specific antibody (BioXCell, Lebanon, NH, USA) was used during the clinically relevant ICI treatment protocol. This ICI treatment is comparable to human ICI treatment regarding effectiveness and potential side effects [4,24]. The PD-1 specific antibody was diluted in PBS (Gibco, Waltham, MA, USA) until a clinically relevant dosage of 200 µg per mouse was attained [4,6,25]. Starting in week 1 of the exercise protocol (see Section 2.3), mice were treated with anti-PD-1 immunotherapy (200 µg/mouse; ICI groups) intraperitoneally twice a week during the 4-week protocol [6,25]. Non-ICI groups received sham saline injections to control for injection-associated stressors.

### 2.3. Low-Intensity Treadmill Exercise Protocol

Mice were divided into sedentary (SED) and low-intensity treadmill exercise groups (TM). Mice in TM groups underwent a low-intensity treadmill exercise protocol 5 days a week for a total of 4 weeks. The protocol was progressive in nature (increasing duration or incline each week) to represent an exercise intervention comparable to clinical exercise interventions [26]. TM groups started with an acclimation period consisting of 20 min treadmill running, starting with a low walking speed (5–8 m/min), and increasing to the specific speed of the protocol (10 m/min), on three days during the week prior to the start of the treadmill protocol.

During the first week of the protocol, mice ran at an incline of zero and speed of 10 m/min for 45 min over 5 days. In week 2, incline and speed stayed the same, but the duration was increased to 60 min per session over 5 days. In week 3 and 4, mice ran at an incline of 2 and a speed of 10 m/min for 60 min per session for 5 days per week (Table 1). Mice performed daily exercise during their active phase (dark cycle) to maximize performance and decrease sleep/behavior disruptions. All mice in TM groups successfully maintained compliance throughout the 4-week exercise protocol and therefore all mice were included in further statistical analyses. SED mice were placed in the same room while TM mice were performing treadmill exercise to control for environmental stressors unrelated to exercise.

### 2.4. Cardiac Structure and Function

Conscious echocardiography (GE Vivid 7 Dimension; Wauwatosa, WI, USA) was used to determine if ICI-treatment induces changes in cardiac structure and function and if low-intensity exercise was able to preserve cardiac structure and function. At baseline and sacrifice timepoints, mice were restrained in a supine position and hair was removed from the chest by means of depilatory agent. Warm transduction jelly was applied to the chest. M-mode images were recorded in the parasternal long-axis view at the level of the papillary muscle to determine left ventricular structure and function. Measurements represented the average of three cardiac cycles from each mouse. UltraLinq software (v2.8.3) was used to analyze the data.

### 2.5. Sacrifice and Tissue Harvest

At the end of the 4-week study, mice were sacrificed at least 24 h after the last treadmill exercise session to evaluate basal biological marker levels. SED mice were sacrificed at the same time. Mice were euthanized via isoflurane overdose followed by cervical crush. Cardiac tissues were excised, flash frozen in liquid nitrogen, or embedded in optimal cutting temperature (OCT) gel and frozen on dry ice. All samples were stored at −80° Celsius until further analysis.

### 2.6. Western Blotting

Upon preparation, approximately 35 mg of cardiac tissue per mouse was homogenized via 8 M Urea lysis buffer with a Qiagen TissueLyser LT homogenizer (Hilden, Germany). Homogenates were centrifuged at 4° Celsius for 10 min at 125,000 G and the supernatant was collected. To determine total protein content in each sample prior to Western Blot analyses, the Bradford Protein Assay was performed [27]. From the Bradford Assay results, equal amounts of protein per sample were used to make Western Blot samples. These samples were loaded into separate wells of a 12-well 4–12% Bis-Tris graded gel (ThermoFisher; Waltham, MA, USA) in addition to MagicMark XP Western Protein Standard (ThermoFisher; Waltham, MA, USA) as a molecular weight comparison. NuPAGE MES or MOPS running buffer (ThermoFisher; Waltham, MA, USA) in a mini-gel NuPAGE gel apparatus (ThermoFisher; Waltham, MA, USA) were used to perform gel electrophoresis. Proteins were then transferred onto a PVDF (polyvinylidene fluoride). PVDF membranes were then blocked in 5% non-fat milk and protein contents were determined by using antibodies against metabolic pathways (anti-AKT, 1:1000 dilution in 5% milk [CST9271]; anti-P-AKT, 1:1000 dilution in 5% milk [CST4060]; anti-FoxO1, 1:500 dilution in 5% milk [CST2880]; anti-FoxO3a, 1:500 dilution in 5% milk [CST12829]; anti-P-FoxO1/3a, 1:500 dilution in 5% milk [CST9464]). Primary antibodies were prepared in 10 mL of 5% non-fat milk solution, at 1:1000 to 1:250 concentration dilution. Membranes were incubated in primary antibody overnight at 4 °C, followed by incubation with a species-specific secondary antibody for 60 min at room temperature. Membranes were developed in ECL (GE Healthcare, RPN2235; GE HealthCare Technologies; Chicago, IL, USA) and imaged via Bio-Rad Chem-Doc XRS+ (Hercules, CA, USA). Protein content was quantified by densitometry (Quantity One, Bio Rad; Hercules, CA, USA), expressed in arbitrary units, and normalized to control GAPDH (anti-GAPDH, 1:4000 dilution in 5% milk [G8795]) protein content of each sample.

### 2.7. Autophagy

RFP (red fluorescent protein)–GFP (green fluorescent protein)–LC3 transgenic mice (C57BL/6-Tg(CAG-RFP/EGFP/Map1lc3b)1Hill/J; Jackson Laboratory strain #027139) were used in this study. These mice express dual tagged LC3 proteins making it possible to analyze autophagic flux in cardiac muscle tissue by means of fluorescent microscopy. As the LC3 protein groups to form an early-phase autophagosome, the RFP (red) and GFP (green) fluorescently tagged proteins will appear as yellow puncta via fluorescent microscopy. As late stage autophagolysosomes are formed, the less stable GFP (green) signal is quenched by the acidic hydrolases from the lysosome, leaving behind only the RFP signal which appears as red puncta via fluorescent microscoy [28]. Cardiac muscle tissue embedded in OCT was cut into 8–12 μm thick slices and mounted on slides with Prolong Diamond Antifade (cat. no. P36961; Invitrogen, Carlsbad, CA, USA) to preserve the tissue. Images of sample slides were obtained by using confocal fluorescent microscopy. Images were taken at 10 random fields of the fixed tissue sample at 40× (EVOS FL Inverted Microscope; ThermoFisher; Waltham, MA, USA). Excitation was set at 488 nm (GFP), 543 nm (RFP), and 405 nm (DAPI). Green and Red Puncta Colocalization Macro for ImageJ (v1.54) [29] was used to objectively analyze autophagosome and autolysosome content in each sample represented by yellow and red puncta.

### 2.8. Tumor Protocol

**Figure 1:**
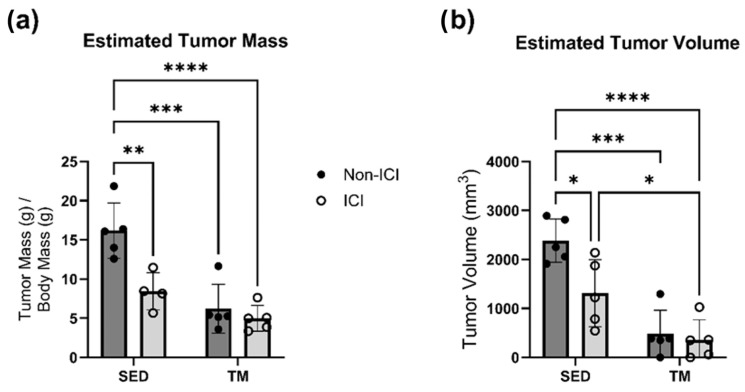
Tumor Characteristics. Tumor characteristics (**a**,**b**). The following formula was used to calculate estimated tumor volume based on caliper measurements (a = longest diameter, b = shortest diameter of the tumor): tumor volume (mm^3^) = (a × b)2/2. SED, sedentary control group; SED + ICI, sedentary ICI-treated group; TM, treadmill-exercised group; TM + ICI, treadmill-exercised ICI-treated group. Values are reported as mean ± SD. * Significant difference, *p* < 0.05; ** significant difference, *p* < 0.01; *** significant difference, *p* < 0.001; **** significant difference, *p* < 0.0001.

As a proof-of-concept to determine efficacy of the administered ICI administration protocol, additional tumor-bearing groups were injected with ICI treatment. Additional animals were randomly selected and separated into four additional groups: sedentary tumor-bearing non-ICI, sedentary tumor-bearing ICI-treated, low-intensity exercised non-ICI, and low-intensity exercised ICI-treated groups. Mice in these tumor-bearing groups were injected with 5 × 10^5^ Lewis Lung Carcinoma cells in the right flank on day 0 (48 h prior to the start of week 1 of the exercise and ICI protocol) and then followed the same exercise and/or ICI treatment protocols for 4 weeks. However, these mice were excluded from all statistical analyses, except to show relevance and efficacy of ICI administration in Figure 1.

### 2.9. Statistical Analyses

Power analyses were performed to detect differences in cardiac structure and function between groups on preliminary data at an alpha level of 0.05 set a priori according to current standards of significance. Shapiro–Wilk tests were performed and did not show evidence of non-normality for all variables. Based on the outcome of the normality testing and after visual examination of QQ plots, further parametric statistical tests were performed. All data is presented as mean ± SD. Statistical software GraphPad Prism (v10; La Jolla, CA, USA) was used for analyses. One mouse in the TM group died prematurely due to unknown circumstances and was therefore excluded from all statistical analyses. No known exercise or ICI injection/treatment-related complications occurred. To address the effects of low-intensity exercise on cardiac structural and functional changes, two-way ANOVA was used to analyze differences between sedentary controls (SED), sedentary ICI-treated mice (SED + ICI), low-intensity exercised controls (TM), and low-intensity exercised ICI-treated mice (TM + ICI). Two-way repeated measures ANOVA was performed to compare baselines to sacrifice measures of the cardiac function within groups. If a significant difference (*p* < 0.05) was identified, Tukey’s post hoc testing was performed to identify where these significant differences occurred. Change scores were performed to calculate percent change in body weight and cardiac function from baseline to sacrifice. To address the effects of low-intensity exercise on cardiac metabolism and autophagy, two-way ANOVA was used to analyze the differences between sedentary controls (SED), sedentary ICI-treated mice (SED + ICI), low-intensity exercised controls (TM), and low-intensity exercised ICI-treated mice (TM + ICI). If a significant difference (*p* < 0.05) was identified, Tukey’s post hoc testing was performed to identify where these significant differences occurred. All analyses were two-tailed, with an alpha level of 0.05 to define statistical significance.

## 3. Results

### 3.1. Efficacy of ICI and Low-Intensity Exercise Protocols

While the focus of this study was to determine the cardioprotective effects of low-intensity exercise on ICI-induced cardiotoxicity, ICI treatment was also administered to tumor-bearing animals as a proof-of-concept of the clinical relevance and efficacy of the administered ICI protocol and the administered low-intensity treadmill exercise protocol. ICI treatment was administered to ICI groups twice per week throughout all 4 weeks of the study (see Section 2.2 above). Two-way ANOVA was performed to analyze the effect of ICI treatment (non-ICI vs. ICI) and activity level (SED vs. TM) on tumor burden (i.e., estimated tumor mass and estimated tumor volume). Two-way ANOVA revealed that there was a statistically significant interaction between the effects of ICI treatment and activity level for analyses of estimated tumor mass (F(1, 15) = 6.418, *p* = 0.0229). Simple main effects analysis revealed that there was a statistically significant effect of both ICI treatment and activity level on estimated tumor mass (*p* < 0.05; Figure 1a). Tukey HSD post hoc tests showed that estimated tumor mass was significantly lower in both ICI groups and both TM groups compared to SED controls (*p* < 0.05; Figure 1a). Two-way ANOVA also revealed that there was not a statistically significant interaction between the effects of ICI treatment and activity level for analyses of estimated tumor volume (F(1, 16) = 4.230, *p* = 0.0564). Simple main effects analysis revealed that there was a statistically significant effect of both ICI treatment and activity level based on estimated tumor volume (*p* < 0.05; Figure 1b). Tukey HSD post hoc tests showed that estimated tumor volume was significantly lower in the SED + ICI group and both TM groups compared to SED controls (*p* < 0.05; Figure 1b). Additionally, TM + ICI mice showed significantly smaller tumor volume compared to SED + ICI mice (*p* < 0.05; Figure 1b).

### 3.2. Low-Intensity Exercise Protects Against ICI Treatment-Induced Cardiac Remodeling and Dysfunction

**Figure 2:**
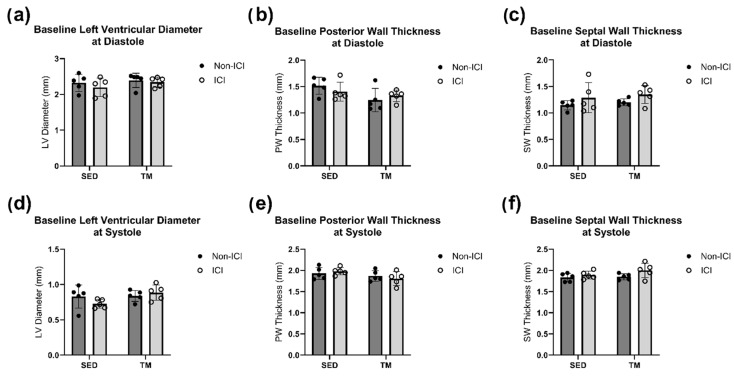
Baseline Geometry. Baseline in vivo echocardiography. An average of three consecutive cardiac cycles per mouse are representative of the wall thicknesses (**b**,**c**,**e**,**f**) and left ventricular diameter (**a**,**d**) at end-diastole and end-systole. No significant differences between study groups for all baseline cardiac measurements were noted at the baseline timepoint.

A low-intensity treadmill exercise protocol was implemented to identify if low-intensity exercise can protect the heart of ICI-treated mice against cardiac dysfunction and remodeling. TM and TM + ICI mice underwent a 4-week low-intensity exercise protocol. In vivo conscious echocardiography measurements were performed at baseline and sacrifice timepoints. Two-way ANOVA was performed to analyze the effect of ICI treatment (non-ICI vs. ICI) and activity level (SED vs. TM) on cardiac geometry measures (i.e., SW thickness, PW thickness, LVD) at systole and diastole. No significant differences in cardiac geometry at baseline were observed between all four groups (Figure 2a–f).

**Figure 3:**
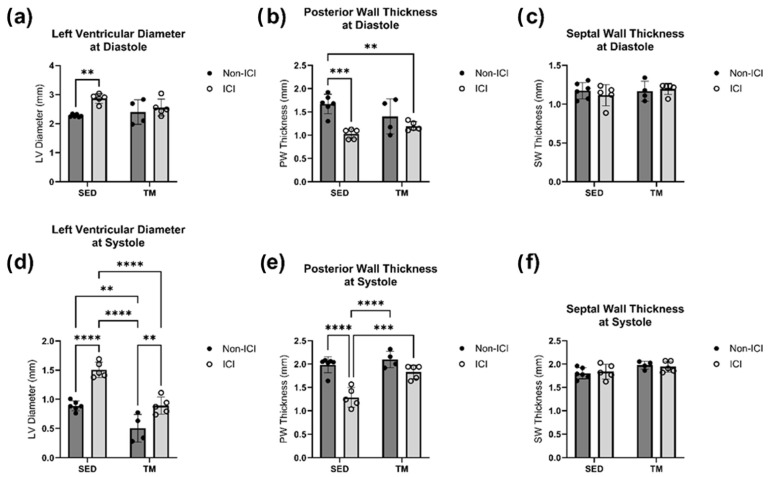
Sacrifice Geometry. In Vivo Echocardiography at Study Endpoint. An average of three consecutive cardiac cycles per mouse are representative of the wall thicknesses (**b**,**c**,**e**,**f**) and left ventricular diameter (**a**,**d**) at end-diastole and end-systole. SED, sedentary control group; SED + ICI, sedentary ICI-treated group; TM, treadmill-exercised group; TM + ICI, treadmill-exercised ICI-treated group. Values are reported as mean ± SD. ** significant difference, *p* < 0.01; *** significant difference, *p* < 0.001; **** significant difference, *p* < 0.0001.

No significant differences at sacrifice in either SWs or SWd were detected between all four groups (Figure 3c,f). However, two-way ANOVA revealed that there were statistically significant interactions between the effects of ICI treatment and activity level for analyses of PW at systole (F(1, 16) = 7.179, *p* = 0.0165) and diastole (F(1, 16) = 5.077, *p* = 0.0386). Simple main effects analysis revealed that there was a statistically significant effect of both ICI treatment and activity level on PW thickness at systole and diastole (*p* < 0.05; Figure 3b,e). Tukey HSD post hoc tests showed that PW thickness was significantly lower in ICI mice compared to SED controls at diastole (*p* < 0.05; Figure 3b) and significantly lower in SED + ICI mice compared to all other groups at systole (*p* < 0.05; Figure 3e). Two-way ANOVA also revealed that there was not a statistically significant interaction between the effects of ICI treatment and activity level on LVD at systole (F(1, 16) = 3.033, *p* = 0.1008) and at diastole (F(1, 16) = 3.827, *p* = 0.0681). Simple main effects analysis showed that both treatment (*p* < 0.05) and activity level (*p* < 0.05) did have a statistically significant effect on LVD at systole (Figure 3d) and that treatment level did have a statistically significant effect on LVD at diastole (Figure 3a). SED + ICI mice showed significantly greater sacrifice LVD measurements at diastole compared to SED controls (*p* < 0.05; Figure 3a) and significantly greater sacrifice LVD measurements at systole compared to all other groups (*p* < 0.05; Figure 3d). While TM + ICI mice showed significantly greater LVD measurements at systole compared to TM controls without ICI treatment, TM + ICI also showed significantly smaller LVD measurements at systole compared to SED + ICI mice, indicating some protection of the cardiac muscle by low-intensity exercise against ICI-induced cardiac remodeling.

**Figure 4:**
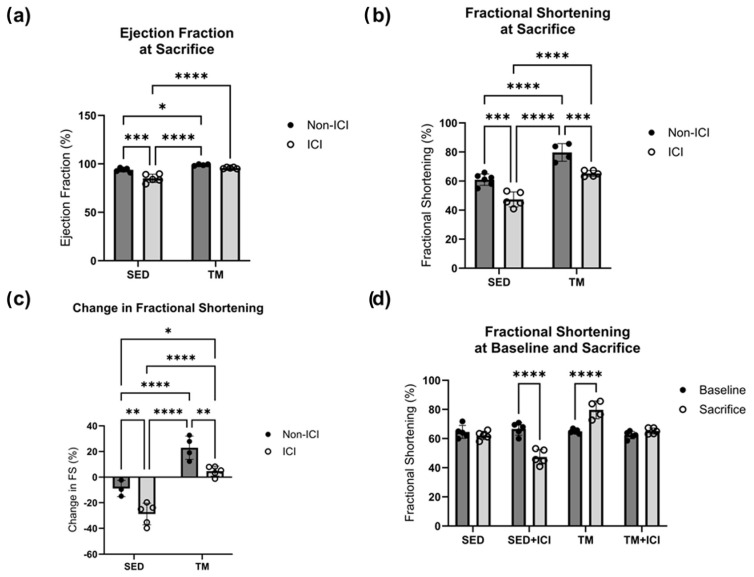
Cardiac Function. Cardiac function at baseline and study endpoint. An average of three consecutive cardiac cycles per mouse represents ejection fraction (**a**) and fractional shortening (**b**) as a measure of cardiac function. Change in cardiac function over the course of the study is represented as change in fractional shortening (**c**), and comparison of fractional shortening at baseline and sacrifice (**d**). SED, sedentary control group; SED + ICI, sedentary ICI-treated group; TM, treadmill-exercised group; TM + ICI, treadmill-exercised ICI-treated group. Values are reported as mean ± SD. * Significant difference, *p* < 0.05; ** significant difference, *p* < 0.01; *** significant difference, *p* < 0.001; **** significant difference, *p* < 0.0001.

Two-way ANOVA was also performed to analyze the effect of ICI treatment (non-ICI vs. ICI) and activity level (SED vs. TM) on cardiac function (i.e., ejection fraction, fractional shortening). No significant differences in cardiac function between all four groups were observed at baseline. Two-way ANOVA for ejection fraction at sacrifice revealed that there was a statistically significant interaction between the effects of ICI treatment and activity level (F(1, 15) = 5.513, *p* = 0.033). Simple main effects analysis revealed that there was a statistically significant effect of both ICI treatment and activity level based on ejection fraction at sacrifice (*p* < 0.05; Figure 4a). Tukey HSD post hoc tests showed that ejection fraction was significantly lower in SED + ICI mice compared to all other groups (*p* < 0.05; Figure 4a). For fractional shortening at sacrifice, a two-way ANOVA revealed that there was not a statistically significant interaction between the effects of treatment and activity level (F(1, 16) = 0.04524, *p* = 0.8343). Simple main effects analysis showed that both ICI treatment (*p* < 0.05) and activity level (*p* < 0.05) did have a statistically significant effect on fractional shortening at sacrifice (Figure 4b). First, TM groups (non-ICI and ICI) experienced significantly greater fractional shortening as a measure of cardiac function compared to SED counterparts (*p* < 0.05; Figure 4b), indicating that while exercise was of low-intensity it was still sufficient to induce exercise-mediated improvements in cardiac function. In line with this, TM control mice were also the only mice who experienced significant increases in cardiac function from baseline to sacrifice compared to all other groups (*p* < 0.05; Figure 4d). Additionally, SED + ICI mice experienced significantly worse fractional shortening at sacrifice compared to all other groups (*p* < 0.05; Figure 4b). While TM + ICI mice showed worse fractional shortening compared to TM controls, cardiac function of TM + ICI at sacrifice was significantly greater compared to SED + ICI counterparts, providing evidence for exercise-mediated cardioprotection to ICI-induced cardiotoxicity. In line with these findings, SED + ICI was the only group that experienced a significant decline in fractional shortening from baseline to sacrifice, based on two-way repeated measures ANOVA (*p* < 0.05; Figure 4d). SED + ICI mice also experienced the greatest negative percent loss in fractional shortening from baseline to sacrifice compared to all other groups (*p* < 0.05; Figure 4c). These data indicate that low-intensity exercise preserved cardiac structure and function in TM + ICI mice comparable to SED levels. Therefore, these data suggest that low-intensity exercise might have the potential to protect the heart against ICI-induced cardiac dysfunction and remodeling.

### 3.3. Low-Intensity Exercise Regulates ICI-Induced Metabolic and Autophagic Dysfunction

**Figure 5:**
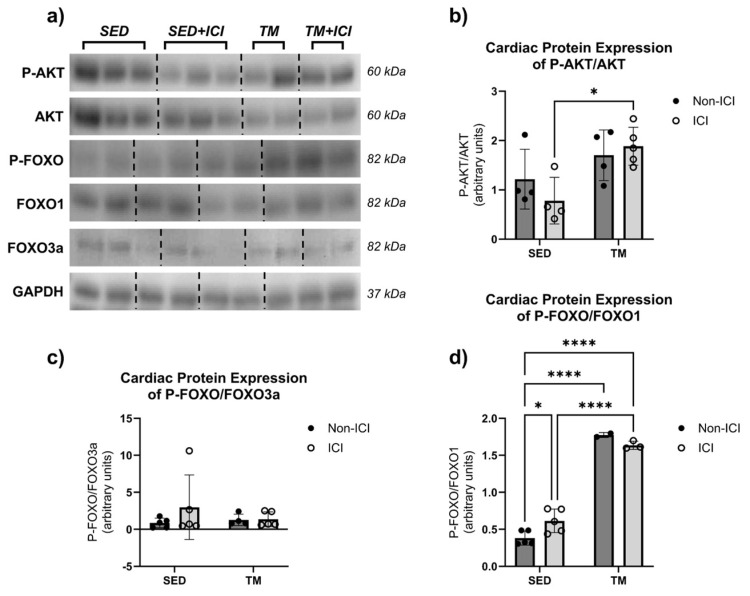
Metabolic Protein Levels. Effects of ICI treatment and low-intensity exercise on cardiac protein levels: representative blot images containing n = 2–3 per group (**a**), P-AKT/AKT (**b**), P-FOXO/FOXO3a (**c**), and P-FOXO/FOXO1 (**d**). SED, sedentary control group; SED + ICI, sedentary ICI-treated group; TM, treadmill-exercised group; TM + ICI, treadmill-exercised ICI-treated group. Values are reported as mean ± SD. * Significant difference, *p* < 0.05; **** significant difference, *p* < 0.0001. The uncropped blots are shown in Figure S1.

Metabolic proteins AKT, FoxO1, and FoxO3a are involved in the control of protein synthesis and protein degradation and therefore play a key role in protein homeostasis (Figure 5a–d). Total protein expression of AKT, FoxO1, and FoxO3a was normalized to GAPDH and did not show significant differences between groups. The phosphorylated isoforms of AKT (i.e., active P-AKT) and FoxO1 and FoxO3a (i.e., inactive P-FOXO) were analyzed in cardiac tissue and normalized to respective total protein expression (i.e., P-AKT/AKT, P-FOXO/FoxO1, P-FOXO/FoxO3a).

For P-AKT levels, two-way ANOVA revealed that there was not a statistically significant interaction between the effects of treatment and activity level on cardiac levels of P-AKT/AKT (F(1, 13) = 1.65, *p* = 0.2213). Simple main effects analysis revealed that there was a statistically significant effect of activity level on P-AKT/AKT levels in cardiac muscle (*p* < 0.05). P-AKT/AKT protein levels were significantly upregulated in the TM + ICI group compared to the SED + ICI group (Figure 5b). These data indicate that, in this model, low-intensity exercise was able to protect the heart from ICI-induced metabolic changes in AKT signaling via upregulation of phosphorylation and activation of AKT, a protein associated with cell survival and growth.

Two-way ANOVA was also performed to analyze the effect of ICI treatment and activity level on cardiac levels of P-FOXO/FoxO1 and P-FOXO/FoxO3a. Two-way ANOVA revealed that there was not a statistically significant interaction between the effects of treatment and activity level on cardiac levels of P-FOXO/FoxO3a (F(1, 15) = 0.8627, *p* = 0.3677; Figure 5c). Simple main effects analysis did not show a statistically significant effect of either treatment or activity level on cardiac FoxO3a levels. However, two-way ANOVA revealed that there was a statistically significant interaction between treatment and activity level on P-FOXO/FoxO1 protein levels in cardiac tissue (F(1, 11) = 8.516, *p* = 0.0140; Figure 5d). Additionally, simple main effects analysis showed that activity level did have a statistically significant effect on P-FOXO/FoxO1 protein levels in cardiac muscle (F(1, 11) = 361.7, *p* < 0.0001). SED + ICI showed significantly greater P-FOXO/FoxO1 levels compared to SED controls (*p* < 0.05). Additionally, both TM groups showed significantly greater P-FOXO/FoxO1 levels compared to SED controls (*p* < 0.05), and TM + ICI showed significantly greater P-FOXO/FoxO1 levels compared to SED + ICI (*p* < 0.05). In our model, these data indicate that ICI treatment significantly upregulated P-FOXO/FoxO1 protein levels in cardiac muscle. However, low-intensity exercise led to an even greater upregulation of P-FOXO/FoxO1 protein levels.

**Figure 6:**
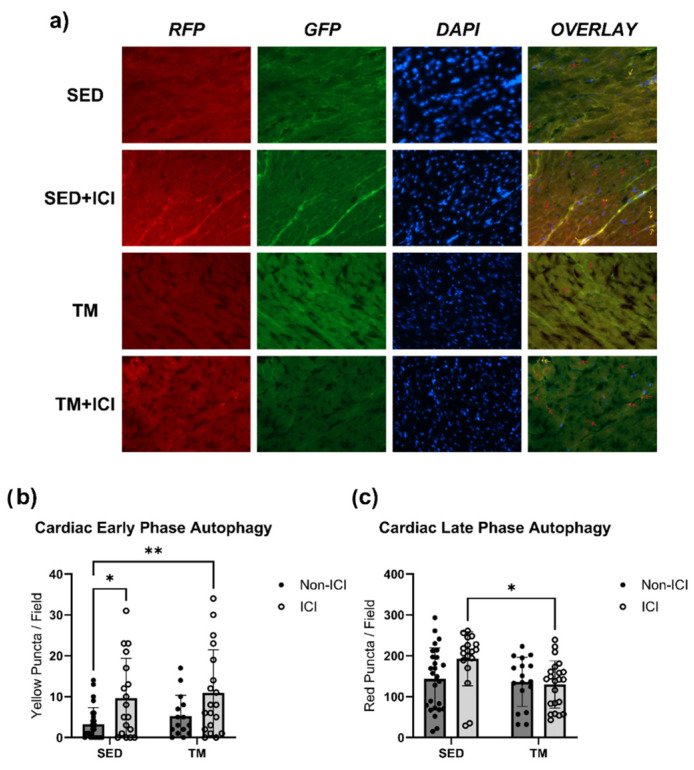
Autophagy. Fluorescent microscopy analysis of autophagic flux in cardiac tissue. Early-phase autophagosomes are represented as yellow puncta (**b**) and late-phase autolysosomes are represented as red puncta (**c**). Representative fluorescent images were taken at 40× magnification (**a**). Yellow and red arrows point towards representative puncta/stages of autophagy. SED, sedentary control group; SED + ICI, sedentary ICI-treated group; TM, treadmill-exercised group; TM + ICI, treadmill-exercised ICI-treated group; RFP, red fluorescent protein; GFP, green, fluorescent protein; DAPI, 4′,6-diamidino-2-phenylindole. Values are reported as mean ± SD. * Significant difference, *p* < 0.05; ** significant difference, *p* < 0.01.

Finally, cardiac muscle tissue of LC3 transgenic mice in SED and TM groups was assessed for the early- and late-phase autophagic puncta via fluorescent microscopy (Figure 6a–c). For early-phase autophagy yellow puncta (Figure 6b), two-way ANOVA revealed that there was not a statistically significant interaction between treatment and activity level on yellow puncta expression in cardiac muscle (F(1, 75) = 0.03504, *p* = 0.8520). Simple main effects analysis showed that there was a statistically significant effect of ICI treatment on yellow puncta expression in cardiac muscle (*p* < 0.05). Expression of yellow puncta associated with early-phase autophagy was significantly greater in both ICI groups (SED + ICI; TM + ICI) compared to SED controls (*p* < 0.05; Figure 6a,b), indicating that ICI treatment significantly increases early-phase autophagy in the heart.

For red puncta expression associated with late-phase autophagy (Figure 6c), two-way ANOVA also revealed that there was not a statistically significant interaction between treatment and activity level on red puncta expression in cardiac muscle (F(1, 79) = 3.606, *p* = 0.0612). Simple main effects analysis showed that there was a statistically significant effect of activity level on red puncta expression in the heart (*p* < 0.05). TM + ICI mice showed significantly lower expression of red puncta, associated with late-phase autophagy, in cardiac muscle compared to SED + ICI mice (*p* < 0.05; Figure 6c). These data indicate that while ICI treatment significantly increases early-phase autophagy in the heart, low-intensity exercise seems to attenuate ICI-induced upregulation of late-phase autophagy. Exercise-mediated protection against ICI-induced upregulated autophagy is likely one mechanism contributing to the protection of cardiac structure and function observed via in vivo echocardiography.

## 4. Discussion

While pharmacological anti-inflammatory treatments have been successful in overcoming ICI-induced cardiotoxicity in a preclinical model [4], aerobic exercise is a promising non-pharmacological alternative that offers a multitude of benefits, including cardioprotective effects which has been observed in both preclinical cancer models and in cancer patients [6,7,8,9]. Studies have shown that exercise can protect against chemotherapy-induced cardiotoxicity by downregulation of inflammation and muscle wasting, attenuation of abnormal cardiac remodeling, and ultimately preserving cardiac function [20,30,31,32,33,34]. While most studies have focused on high-intensity exercise or moderate-to-high-intensity exercise only, many cancer patients prefer low- or mild-intensity exercise that they can participate in without medical supervision [14,35]. However, clinical and preclinical research focusing on low-intensity exercise effects on cancer, anti-cancer treatment efficacy, and protection against anti-cancer treatment-induced side effects remain understudied. Determining the benefits of low-intensity exercise as an adjuvant anti-cancer treatment strategy is an urgent need as it could significantly improve adherence and compliance to exercise programs as part of cancer treatment regimens, increasing the range of cancer patients benefiting from the protective effects of exercise.

While there is a growing body of research investigating the protective effects of exercise against chemotherapy-induced cardiotoxicity, to our knowledge, the effects of any modality of exercise, and specifically low-intensity exercise, against ICI-induced cardiotoxicity have yet to be determined. Therefore, this study sought to identify cardioprotective effects of a 4-week low-intensity treadmill exercise protocol against ICI-induced changes in cardiac structure, function, and underlying molecular signaling pathways in mice. The specific low-intensity exercise protocol was chosen due to its ability to protect cardiac muscle tissue against cancer-mediated cardiac cachexia in the same mouse model as used in this study [36].

In our study, low-intensity exercise was capable of significantly protecting cardiac structures against ICI-induced atrophy of the ventricular walls and significantly preserved cardiac function after 4 weeks of ICI treatment. While TM mice without ICI treatment showed significant improvements in cardiac function above resting levels, indicative of muscular adaptations due to low-intensity exercise, TM + ICI mice showed preservation of cardiac structure and function similar to the levels of non-ICI-treated SED controls. Therefore, our low-intensity exercise protocol successfully protected cardiac structure and function against ICI-induced remodeling and dysfunction, comparative to previously determined exercise benefits against chemotherapy-induced cardiotoxicity [37,38,39].

Metabolism and proteolytic autophagic pathways were affected by ICI and low-intensity exercise. In chemotherapy-induced cardiotoxicity, aerobic exercise has been shown to regulate metabolic and muscle wasting pathways via attenuation of proteolytic systems [40]. Research has shown that doxorubicin chemotherapy treatment activates FoxO signaling pathways and therefore increases proteolytic systems downstream [41,42,43]. Activation of FoxO1 and FoxO3a was significantly reduced following acute aerobic exercise and prolonged endurance exercise accompanied by exercise-mediated protection against chemotherapy-induced cardiac atrophy and myotoxicity [44,45]. However, the functions of FoxO pathways and the post-translational modifications and associated changes in the function of FoxO in the nucleus and cytosol are complex. In our study, both exercised groups (TM and TM + ICI) experienced significant upregulation in phosphorylated FoxO1 protein expression compared to both SED groups. Interestingly, in vivo and in vitro research under healthy physiological conditions has shown that FoxO1 is required for normal cardiac adaptations to aerobic exercise [46]. Therefore, upregulation of FoxO1 in our model may play a cardioprotective role.

In agreement with this are the findings of upregulated activation of AKT signaling pathways in the TM mice of our study. Exercise has been shown to increase activity of the AKT pathway and induce cardiac protection via anti-apoptotic, angiogenic, and anti-fibrotic mechanisms [47,48,49,50]. Contrary to chemotherapy-induced cardiotoxicity, low-intensity exercise-mediated upregulation of AKT/FoxO1 in this ICI model may have cardioprotective properties. However, further research is needed to confirm these findings and explore downstream signaling pathways.

FoxO pathways are key regulators of autophagic degradation pathways, either via binding to transcription factors of degradation proteins in the nucleus or interaction with autophagic proteins in the cytosol. Previously, it has been shown that autophagy may play a dual role in the protection of the heart, with moderate levels protecting against cardiac damage, but excessive levels of autophagy accelerating cardiac cell death [51]. Therefore, maintaining homeostasis of autophagic flux is critical for maintenance of cardiovascular function. Abnormally upregulated early-phase autophagy can lead to excessive engulfing of cardiac proteins and organelles, whereas abnormally upregulated late-phase autophagy can lead to excessive decomposing and recycling of organelles, both resulting in disruption of cardiovascular homeostasis and dysfunction [52]. Different moderate-to-high-intensity exercise interventions have been shown to protect against doxorubicin-induced dysfunctions in autophagy by downregulating the expression of autophagosomes in cardiac tissue [38]. ICI-treated mice in this study showed similar findings. SED + ICI mice showed significantly more early-phase and late-phase autophagy compared to SED controls. While TM + ICI mice showed significantly more early-phase autophagosomes compared to SED non-ICI controls, late-phase autophagolysosome expression in TM + ICI mice was not significantly different compared to SED mice and was significantly lower compared to SED + ICI counterparts. These findings are in line with previously noted studies that suggest a cardioprotective role of increased activation of FoxO1 following aerobic exercise that may lead to regulated protein homeostasis in the heart under normal physiological conditions and in response to exercise [46,53,54]. Therefore, while low-intensity exercise did not seem to affect early-phase autophagy (initiation phase), low-intensity exercise was able to protect cardiac tissue from ICI-induced upregulation in late-phase autophagy. These data agree with previous research suggesting cardioprotective effects of exercise against ICI-induced pathophysiological upregulation of degradation pathways.

## 5. Conclusions

In summary, the ICI treatment protocol used in this study led to significant cardiac dysfunction and remodeling, accompanied by underlying dysfunctional metabolism and autophagy. Low-intensity exercise was successful in protecting against ICI-induced cardiotoxicity by preserving cardiac structure and function. Mechanisms that are likely involved in the cardioprotective effects of low-intensity exercise against ICI-induced cardiotoxicity include regulation of metabolism through maintenance of protein homeostasis via upregulation of protein synthesis (i.e., AKT, FoxO1) pathways and downregulation of abnormal protein degradation (i.e., autophagic flux). Therefore, even though of only low intensity, our clinically relevant low-intensity exercise protocol elicited cardioprotective effects against ICI-induced cardiotoxicity. This study adds knowledge to the characterization of still unclear clinical manifestations of ICI-induced cardiotoxicity, underlying signaling pathways that could shed light on potential pharmacological treatment targets, as well as the protective effects of low-intensity exercise as a non-pharmacological treatment strategy.

## Figures and Tables

**Table 1:** Low-Intensity Treadmill Exercise Protocol.

	Week 1	Week 2	Week 3	Week 4
Incline (°)	0	0	2	2
Speed (m/min)	10	10	10	10
Duration (min)	45	60	60	60
Days per Week	5	5	5	5

## Data Availability

The raw data supporting the conclusions of this article will be made available by the authors on request.

## Supplementary Materials

The following supporting information can be downloaded at: https://www.mdpi.com/article/10.3390/cancers18010138/s1, Supplemental Figure S1. Whole Western Blot Images—Cardiac Tissue.

## References

[1] McCluskey K. Immunotherapy vs. Chemotherapy: What’s the Difference? Cancer Research Institute. https://www.cancerresearch.org/blog/difference-cancer-immunotherapy-and-chemotherapy.

[2] Garon E.B., Hellmann M.D., Rizvi N.A., Carcereny E., Leighl N.B., Ahn M.-J., Eder J.P., Balmanoukian A.S., Aggarwal C., Horn L. (2019). Five-Year Overall Survival for Patients with Advanced Non–Small-Cell Lung Cancer Treated with Pembrolizumab: Results from the Phase I KEYNOTE-001 Study. J. Clin. Oncol..

[3] Li C., Bhatti S.A., Ying J. (2022). Immune Checkpoint Inhibitors—Associated Cardiotoxicity. Cancers.

[4] Gergely T., Kucsera D., Toth V., Kovacs T., Sayour N., Drobni Z., Ruppert M., Petrovich B., Agg B., Onodi Z. (2022). Characterization of immune checkpoint inhibitor-induced cardiotoxicity reveals interleukin-17A as a driver of cardiac dysfunction after anti-PD-1 treatment. Br. J. Pharmacol..

[5] Parry T.L., Hayward R. (2018). Exercise Protects Against Cancer-Induced Cardiac Cachexia. Med. Sci. Sports Exerc..

[6] Kurz E., Hirsch C.A., Dalton T., Shadaloey S.A., Khodadadi-Jamayran A., Miller G., Pareek S., Rajaei H., Mohindroo C., Baydogan S. (2022). Exercise-induced engagement of the IL-15/IL-15Rα axis promotes anti-tumor immunity in pancreatic cancer. Cancer Cell.

[7] Martín-Ruiz A., Fiuza-Luces C., Rincón-Castanedo C., Fernández-Moreno D., Martínez-Martínez E., Martín-Acosta P., Coronado M.J., Franco-Luzón L., Ramírez M., Provencio M. (2020). Benefits of exercise and immunotherapy in a murine model of human non–small-cell lung carcinoma. Exerc. Immunol. Rev..

[8] Allan J., Buss L.A., Draper N., Currie M.J. (2022). Exercise in People with Cancer: A Spotlight on Energy Regulation and Cachexia. Front. Physiol..

[9] Parry T.L., Tichy L., Brantley J.T. (2022). Cardioprotective effects of preconditioning exercise in the female tumor bearing mouse. Front. Cell Dev. Biol..

[10] Tichy L., Parry T.L. (2023). The pathophysiology of cancer-mediated cardiac cachexia and novel treatment strategies: A narrative review. Cancer Med..

[11] Rundqvist H., Veliça P., Barbieri L., Gameiro P.A., Bargiela D., Gojkovic M., Mijwel S., Reitzner S.M., Wulliman D., Ahlstedt E. (2020). Cytotoxic T-cells mediate exercise-induced reductions in tumor growth. eLife.

[12] El-Rayes M., Nardi Agmon I., Yu C., Osataphan N., Yu H.A., Hope A., Sacher A., Yu A.F., Abdel-Qadir H., Thavendiranathan P. (2025). Lung Cancer and Cardiovascular Disease. JACC CardioOncol..

[13] Verdicchio C., Freene N., Hollings M., Maiorana A., Briffa T., Gallagher R., Hendriks J.M., Abell B., Brown A., Colquhoun D. (2023). A Clinical Guide for Assessment and Prescription of Exercise and Physical Activity in Cardiac Rehabilitation. A CSANZ Position Statement. Heart Lung Circ..

[14] Wasley D., Gale N., Roberts S., Backx K., Nelson A., van Deursen R., Byrne A. (2018). Patients with established cancer cachexia lack the motivation and self-efficacy to undertake regular structured exercise. Psychooncology.

[15] Gilchrist S.C., Howard V.J., Akinyemiju T., Judd S.E., Cushman M., Hooker S.P., Diaz K.M. (2020). Association of Sedentary Behavior with Cancer Mortality in Middle-aged and Older US Adults. JAMA Oncol..

[16] Phillips S.M., Dodd K.W., Steeves J., McClain J., Alfano C.M., McAuley E. (2015). Physical Activity and Sedentary Behavior in Breast Cancer Survivors: New Insight into Activity Patterns and Potential Intervention Targets. Gynecol. Oncol..

[17] Friedenreich C.M., Ryder-Burbidge C., McNeil J. (2021). Physical activity, obesity and sedentary behavior in cancer etiology: Epidemiologic evidence and biologic mechanisms. Mol. Oncol..

[18] Chicco A.J., Hydock D.S., Schneider C.M., Hayward R. (2006). Low-intensity exercise training during doxorubicin treatment protects against cardiotoxicity. J. Appl. Physiol..

[19] Gomes-Santos I.L., Jordão C.P., Passos C.S., Brum P.C., Oliveira E.M., Chammas R., Camargo A.A., Negrão C.E. (2021). Exercise Training Preserves Myocardial Strain and Improves Exercise Tolerance in Doxorubicin-Induced Cardiotoxicity. Front. Cardiovasc. Med..

[20] Hydock D.S., Lien C.-Y., Jensen B.T., Parry T.L., Schneider C.M., Hayward R. (2012). Rehabilitative exercise in a rat model of doxorubicin cardiotoxicity. Exp. Biol. Med..

[21] Brumberger Z.L., Branch M.E., Klein M.W., Seals A., Shapiro M.D., Vasu S. (2022). Cardiotoxicity risk factors with immune checkpoint inhibitors. Cardio-Oncology.

[22] Addison D., Branch M., Baik A.H., Fradley M.G., Okwuosa T., Reding K.W., Simpson K.E., Suero-Abreu G.A., Yang E.H., Yancy C.W. (2023). Equity in Cardio-Oncology Care and Research: A Scientific Statement from the American Heart Association. Circulation.

[23] Fernández-Ruiz I. (2023). Hormone therapy ameliorates ICI-related myocarditis in mice. Nat. Rev. Cardiol..

[24] Bu M.T., Yuan L., Klee A.N., Freeman G.J. (2022). A Comparison of Murine PD-1 and PD-L1 Monoclonal Antibodies. Monoclon. Antibodies Immunodiagn. Immunother..

[25] Savage H., Pareek S., Lee J., Ballarò R., Conterno Minussi D., Hayek K., Sadullozoda M., Lochmann B.S., McQuade J.L., LaVoy E.C. (2023). Aerobic Exercise Alters the Melanoma Microenvironment and Modulates ERK5 S496 Phosphorylation. Cancer Immunol. Res..

[26] Foulkes S.J., Howden E.J., Haykowsky M.J., Antill Y., Salim A., Nightingale S.S., Loi S., Claus P., Janssens K., Mitchell A.M. (2023). Exercise for the Prevention of Anthracycline-Induced Functional Disability and Cardiac Dysfunction: The BREXIT Study. Circulation.

[27] Kruger N.J. (1994). The Bradford method for protein quantitation. Methods Mol. Biol. Clifton NJ.

[28] Xie M., Morales C.R., Lavandero S., Hill J.A. (2011). Tuning flux: Autophagy as a target of heart disease therapy. Curr. Opin. Cardiol..

[29] Pampliega O., Orhon I., Patel B., Sridhar S., Díaz-Carretero A., Beau I., Codogno P., Satir B.H., Satir P., Cuervo A.M. (2013). Functional interaction between autophagy and ciliogenesis. Nature.

[30] Antunes P., Esteves D., Nunes C., Sampaio F., Ascensão A., Vilela E., Teixeira M., Amarelo A.L., Joaquim A. (2019). Impact of exercise training on cardiotoxicity and cardiac health outcomes in women with breast cancer anthracycline chemotherapy: A study protocol for a randomized controlled trial. Trials.

[31] Ballarò R., Beltrà M., De Lucia S., Pin F., Ranjbar K., Hulmi J.J., Costelli P., Penna F. (2019). Moderate exercise in mice improves cancer plus chemotherapy-induced muscle wasting and mitochondrial alterations. FASEB J..

[32] Díaz-Balboa E., González-Salvado V., Rodríguez-Romero B., Martínez-Monzonís A., Pedreira-Pérez M., Palacios-Ozores P., López-López R., Peña-Gil C., González-Juanatey J.R. (2021). A randomized trial to evaluate the impact of exercise-based cardiac rehabilitation for the prevention of chemotherapy-induced cardiotoxicity in patients with breast cancer: ONCORE study protocol. BMC Cardiovasc. Disord..

[33] Schneider C., Ryffel C., Rabaglio M., Suter T.M., Campbell K.L., Eser P., Wilhelm M. (2023). Supervised exercise training in patients with cancer during anthracycline-based chemotherapy to mitigate cardiotoxicity—A randomized controlled trial. medRxiv.

[34] Goldschmidt S., Schmidt M.E., Rosenberger F., Wiskemann J., Steindorf K. (2024). Patterns and influencing factors of exercise attendance of breast cancer patients during neoadjuvant chemotherapy. Support. Care Cancer.

[35] Avancini A., Pala V., Trestini I., Tregnago D., Mariani L., Sieri S., Krogh V., Boresta M., Milella M., Pilotto S. (2020). Exercise Levels and Preferences in Cancer Patients: A Cross-Sectional Study. Int. J. Environ. Res. Public Health.

[36] Tichy L., Parry T.L. (2025). Low-Intensity Exercise Protects Skeletal and Cardiac Muscle Against Cancer-Mediated Muscle Wasting via Regulation of Autophagy and Inflammation. JCSM Commun..

[37] Naaktgeboren W.R., Binyam D., Stuiver M.M., Aaronson N.K., Teske A.J., van Harten W.H., Groen W.G., May A.M. (2021). Efficacy of Physical Exercise to Offset Anthracycline-Induced Cardiotoxicity: A Systematic Review and Meta-Analysis of Clinical and Preclinical Studies. J. Am. Heart Assoc..

[38] Wang F., Chandra J., Kleinerman E.S. (2021). Exercise intervention decreases acute and late doxorubicin-induced cardiotoxicity. Cancer Med..

[39] Kang D.-W., Wilson R.L., Christopher C.N., Normann A.J., Barnes O., Lesansee J.D., Choi G., Dieli-Conwright C.M. (2022). Exercise Cardio-Oncology: Exercise as a Potential Therapeutic Modality in the Management of Anthracycline-Induced Cardiotoxicity. Front. Cardiovasc. Med..

[40] Gaytan S.L., Lawan A., Chang J., Nurunnabi M., Bajpeyi S., Boyle J.B., Han S.M., Min K. (2023). The beneficial role of exercise in preventing doxorubicin-induced cardiotoxicity. Front. Physiol..

[41] Xia P., Chen J., Liu Y., Fletcher M., Jensen B.C., Cheng Z. (2020). Doxorubicin induces cardiomyocyte apoptosis and atrophy through cyclin-dependent kinase 2–mediated activation of forkhead box O1. J. Biol. Chem..

[42] Hui R.C.-Y., Francis R.E., Guest S.K., Costa J.R., Gomes A.R., Myatt S.S., Brosens J.J., Lam E.W.-F. (2008). Doxorubicin activates FOXO3a to induce the expression of multidrug resistance gene ABCB1 (MDR1) in K562 leukemic cells. Mol. Cancer Ther..

[43] Ho K.-K., McGuire V.A., Koo C.-Y., Muir K.W., de Olano N., Maifoshie E., Kelly D.J., McGovern U.B., Monteiro L.J., Gomes A.R. (2012). Phosphorylation of FOXO3a on Ser-7 by p38 Promotes Its Nuclear Localization in Response to Doxorubicin. J. Biol. Chem..

[44] Kwon I., Go G.-W., Lee Y., Kim J.-H. (2022). Prolonged Endurance Exercise Adaptations Counteract Doxorubicin Chemotherapy-Induced Myotoxicity in Mice. Appl. Sci..

[45] Kavazis A.N., Smuder A.J., Powers S.K. (2014). Effects of short-term endurance exercise training on acute doxorubicin-induced FoxO transcription in cardiac and skeletal muscle. J. Appl. Physiol..

[46] Weeks K.L., Tham Y.K., Yildiz S.G., Alexander Y., Donner D.G., Kiriazis H., Harmawan C.A., Hsu A., Bernardo B.C., Matsumoto A. (2021). FoxO1 is required for physiological cardiac hypertrophy induced by exercise but not by constitutively active PI3K. Am. J. Physiol. Heart Circ. Physiol..

[47] Shiojima I., Walsh K. (2006). Regulation of cardiac growth and coronary angiogenesis by the Akt/PKB signaling pathway. Genes Dev..

[48] Weeks K.L., Bernardo B.C., Ooi J.Y.Y., Patterson N.L., McMullen J.R., Xiao J. (2017). The IGF1-PI3K-Akt Signaling Pathway in Mediating Exercise-Induced Cardiac Hypertrophy and Protection. Exercise for Cardiovascular Disease Prevention and Treatment: From Molecular to Clinical, Part 2.

[49] Kim Y.K., Kim S.J., Yatani A., Huang Y., Castelli G., Vatner D.E., Liu J., Zhang Q., Diaz G., Zieba R. (2003). Mechanism of enhanced cardiac function in mice with hypertrophy induced by overexpressed Akt. J. Biol. Chem..

[50] Liao J., Li Y., Zeng F., Wu Y. (2015). Regulation of mTOR Pathway in Exercise-induced Cardiac Hypertrophy. Int. J. Sports Med..

[51] De Meyer G.R.Y., Martinet W. (2009). Autophagy in the cardiovascular system. Biochim. Biophys. Acta BBA—Mol. Cell Res..

[52] Wang L., Wang J., Cretoiu D., Li G., Xiao J. (2020). Exercise-mediated regulation of autophagy in the cardiovascular system. J. Sport Health Sci..

[53] Yu W., Chen C., Cheng J. (2020). The role and molecular mechanism of FoxO1 in mediating cardiac hypertrophy. ESC Heart Fail..

[54] Cheng Z. (2019). The FoxO–Autophagy Axis in Health and Disease. Trends Endocrinol. Metab..

