# Supplementary material for: Low-Intensity Exercise Attenuates Immune Checkpoint Inhibitor-Induced Cardiotoxicity via Regulation of Metabolism and Autophagy
Source: Cancers (Basel). 2025 Dec 31;18(1):138. doi: 10.3390/cancers18010138 (PMC12784886; doi:10.3390/cancers18010138)

Cancers Journal
Title: *Low-Intensity Exercise Attenuates Immune Checkpoint Inhibitor-Induced Cardiotoxicity via Regulation of Metabolism and Autophagy*
Louisa Tichy & Traci L. Parry

Supplemental Figure S1. Whole Western Blot Images – Cardiac Tissue

1. **AKT**

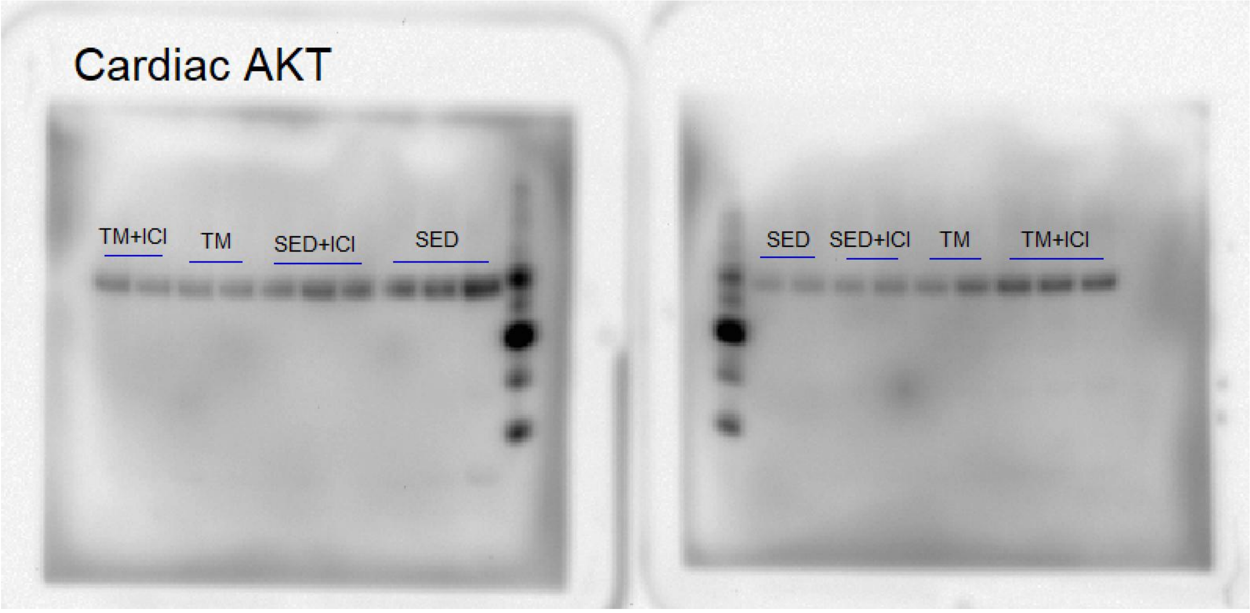

2. **P-AKT**

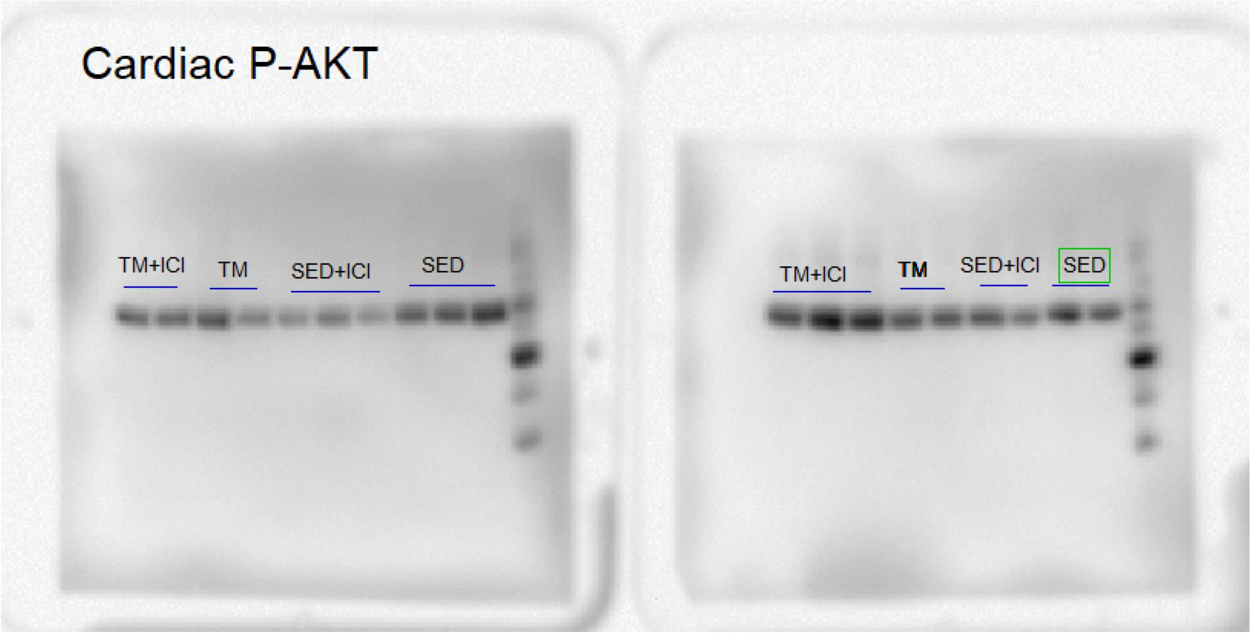

3. **FoxO1**

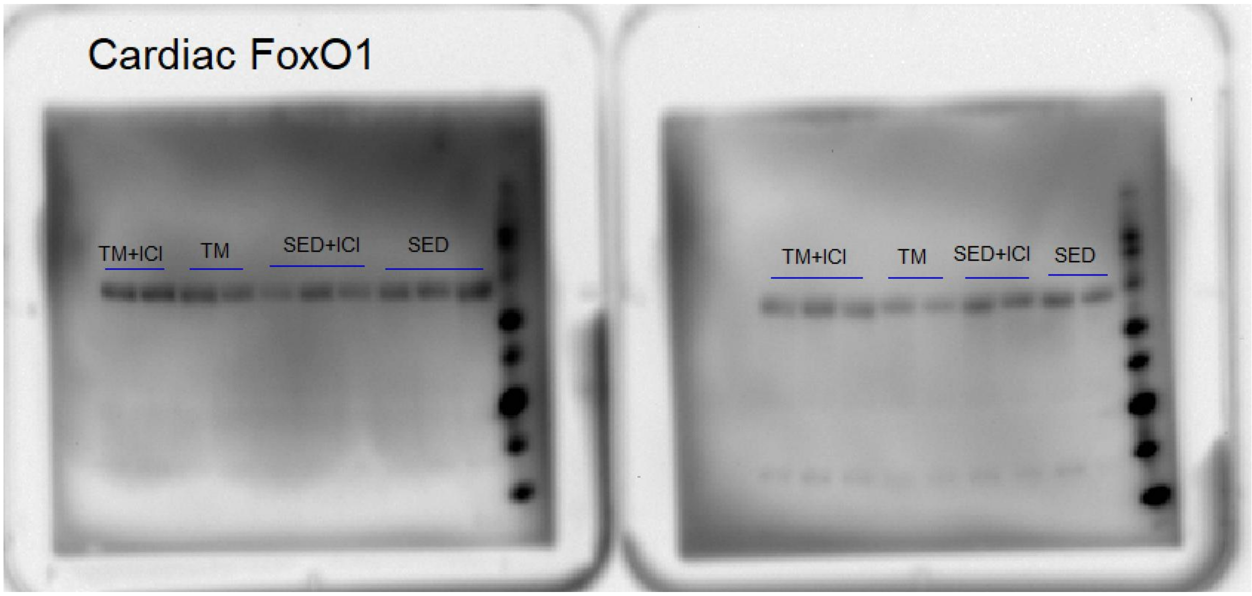

4. **FoxO3a**

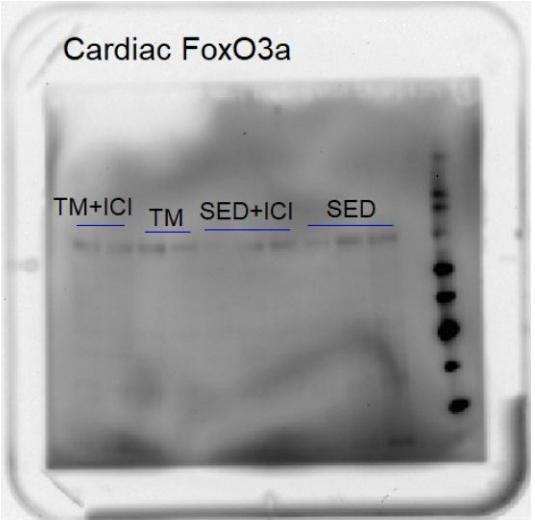

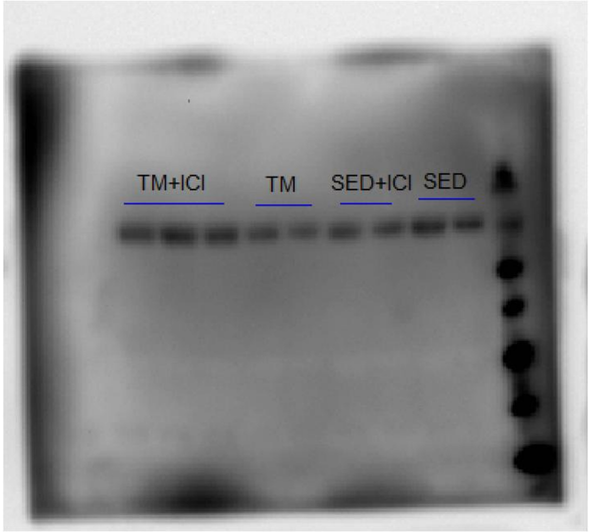

5. P-FOXO

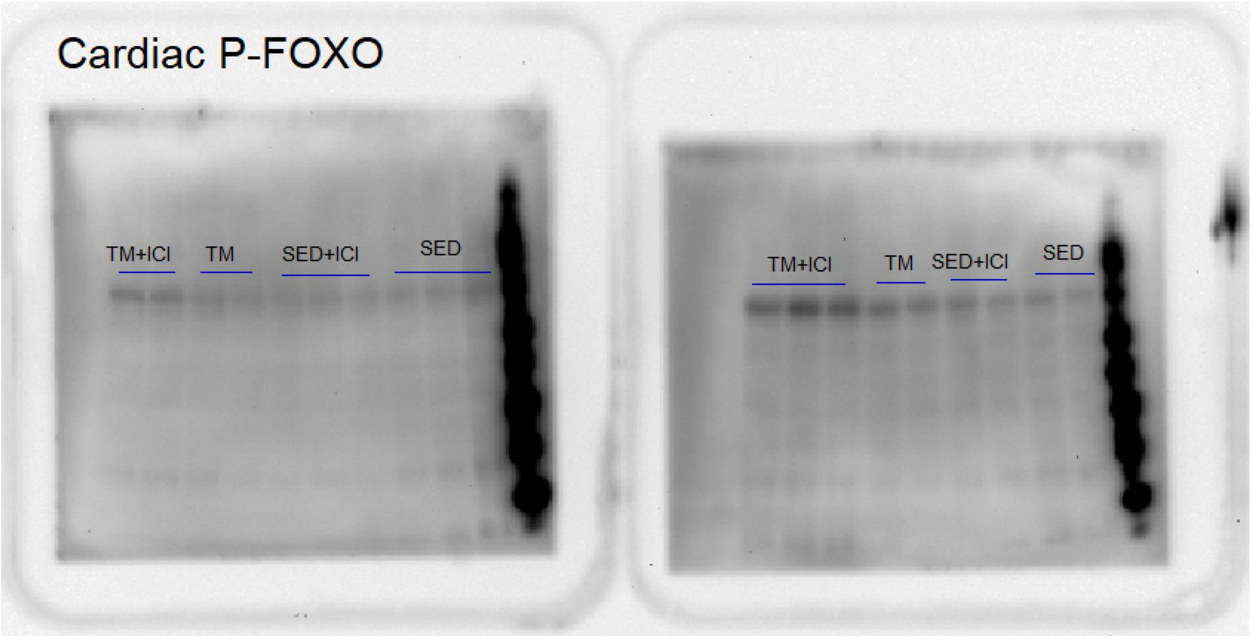

6. GAPDH

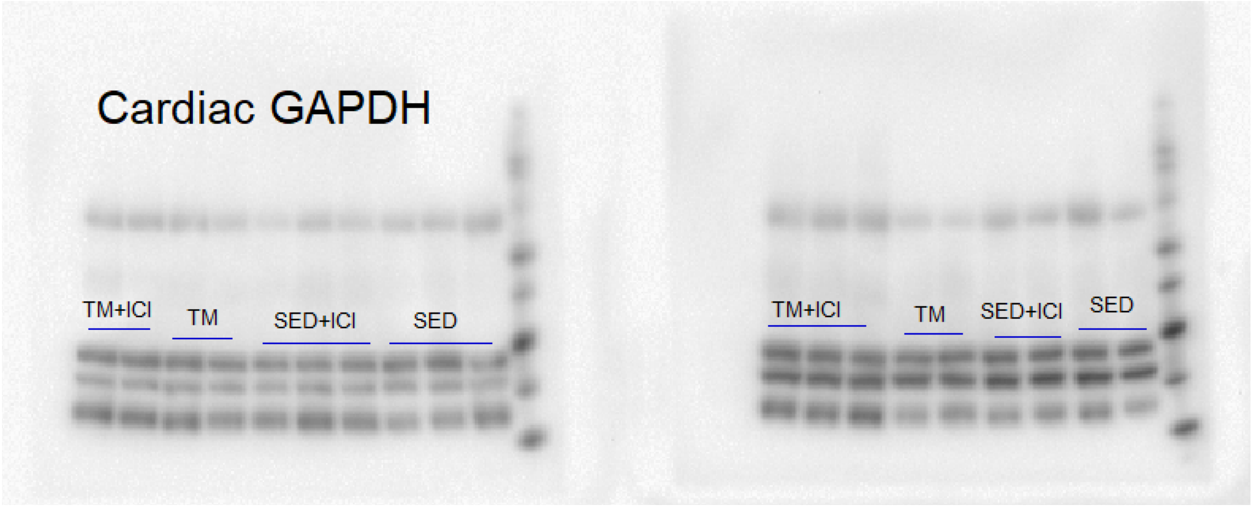

Supplement: Supplementary file 1 [file cancers-18-00138-s001.zip › cancers-4004428-supplementary.pdf]
